# Low Dose of Some Persistent Organic Pollutants Predicts Type 2 Diabetes: A Nested Case–Control Study

**DOI:** 10.1289/ehp.0901480

**Published:** 2010-05-05

**Authors:** Duk-Hee Lee, Michael W. Steffes, Andreas Sjödin, Richard S. Jones, Larry L. Needham, David R. Jacobs

**Affiliations:** 1 Department of Preventative Medicine, School of Medicine, Kyungpook National University, Daegu, Korea; 2 Department of Laboratory Medicine and Pathology, School of Medicine, University of Minnesota, Minneapolis, Minnesota, USA; 3 Organic Analytical Toxicology Branch, Division of Laboratory Sciences, National Center for Environmental Health, Centers for Disease Control and Prevention, Atlanta, Georgia, USA; 4 Division of Epidemiology, School of Public Health, University of Minnesota, Minneapolis, Minnesota, USA; 5 Department of Nutrition, University of Oslo, Oslo, Norway

**Keywords:** diabetes, obesity, organochlorine pesticides, persistent organic pollutants, polychlorinated biphenyls

## Abstract

**Background:**

Low doses of some persistent organic pollutants (POPs) associate cross-sectionally with type 2 diabetes, whereas associations with high POP exposures are inconsistent.

**Objectives:**

We investigated whether several POPs prospectively predict type 2 diabetes within the Coronary Artery Risk Development in Young Adults (CARDIA) cohort.

**Methods:**

Participants in this nested case–control study were diabetes free in 1987–1988. By 2005–2006, the 90 controls remained free of diabetes, whereas the 90 cases developed diabetes. Using serum collected in 1987–1988, we measured 8 organochlorine pesticides, 22 polychlorinated biphenyl congeners (PCBs), and 1 polybrominated biphenyl (PBB). We compared POP concentrations from CARDIA and the National Health and Nutrition Examination Survey (NHANES) in 2003–2004. We computed odds ratios (ORs) for incident diabetes using logistic regression analysis.

**Results:**

Chlorinated POPs in CARDIA in 1987–1988 were much higher than corresponding NHANES 2003-2004 concentrations. POPs showed nonlinear associations with diabetes risk. The highest risk was observed in the second quartiles of *trans*-nonachlor, oxychlordane, mirex, highly chlorinated PCBs, and PBB153—a finding that suggests low-dose effects. We concentrated risk by summing these POPs and isolated very low concentrations of multiple POPs in the lowest sextile of the sum. The adjusted OR in the second sextile vs. the lowest sextile was 5.3 overall and 20.1 for body mass index ≥ 30 kg/m^2^.

**Conclusions:**

Several POPs at low doses similar to current exposure levels may increase diabetes risk, possibly through endocrine disruption. Certain POPs may a play a role in the current epidemic of diabetes, which has been attributed to obesity.

Persistent organic pollutants (POPs) are a group of chemicals with common properties such as persistence, lipophilicity, and biomagnification in the food chain (Li et al. 2006). Lee et al. (2006b, 2007c) recently reported that serum concentrations of POPs were strongly associated with the prevalence of type 2 diabetes in the U.S. general population; this association was stronger among obese persons than among nonobese persons. Although dioxins have been widely studied as the most toxic chemical among POPs, organochlorine (OC) pesticides and polychlorinated biphenyl (PCB) congeners, not dioxins, were strongly associated with type 2 diabetes. In particular, it was striking that when POPs concentrations were very low, prevalent type 2 diabetes was rare even among obese persons [body mass index (BMI) ≥ 30 kg/m^2^]. Serum concentrations of these POPs were also associated with insulin resistance and adverse lipid profiles (Lee et al. 2007a, 2007b). Xenobiotics mainly bioaccumulate in adipose tissue; thus, all these findings raise the possibility that some POPs are critically involved in the pathogenesis of type 2 diabetes (Lee et al. 2006a). However, because all these studies were cross-sectional, reverse causality, in which diabetes enhances POPs accumulation or inhibits their clearance, cannot be ruled out.

One recent study of a cohort of 471 Great Lakes sport fish consumers identified 36 incident cases of type 2 diabetes during follow-up examinations and demonstrated an association of incident diabetes with serum concentrations of 2,2-bis(4-chlorophenyl)-1,1-dichloroethene (*p,p′*--DDE), but not PCBs (Turyk et al. 2009). However, several prospective studies of selected POPS performed in occupational or accidental high-exposure settings reported inconsistent results, particularly for 2,3,7,8-tetrachlorodibenzo-*p*-dioxin (2,3,7,8-TCDD) (Henriksen et al. 1997; Longnecker and Michalek 2000; Steenland et al. 1999). Furthermore, the decreasing trend of OC POPs during recent decades (Needham et al. 2005) is inconsistent with the current trend toward an increased prevalence of type 2 diabetes.

Considering these inconsistencies, we hypothesized that adverse metabolic effects due to POPs may occur more at low but persistent exposures rather than at high exposures, for example, if the adverse effects were caused by endocrine disruption (Lee et al. 2006a). In fact, strong biological effects in low doses, but weak or no effects in higher doses (producing an inverted U-shaped graphic appearance) are proposed as possible biological responses to endocrine disruptors (Daston et al. 2003; Welshons et al. 2003). In this scenario, current background POPs serum concentrations could be even more biologically active than were the higher serum concentrations typically experienced before many POPs were banned. If there were low-dose effects (rapid rise in risk across low doses, with flattened or even attenuated risk at higher doses), the failure to select a true reference group with very low concentrations of POPs would have distorted the true epidemiological associations in high-exposure settings, leading to inconsistent results (Henriksen et al. 1997; Longnecker and Michalek 2000; Steenland et al. 1999). Furthermore, the inverted U-shaped associations with biological outcomes typical of low-dose effects of endocrine disruptors would be missed under the assumption of a linear dose–response relation, which is often taken as a condition for establishing causality in epidemiological studies (Lee et al. 2006a).

We addressed the issues of temporal relation of exposure and disease and of possible low-dose, nonlinear associations in a prospective, nested case–control study of the relationship between background exposure to certain POPs and the development of type 2 diabetes within the Coronary Artery Risk Development in Young Adults (CARDIA) study. Based on previous findings, we also hypothesized that we would find stronger associations with summary POPs measures than with individual POPs and that these associations would be stronger among obese persons than among nonobese persons.

## Materials and Methods

### Source population

CARDIA cohort study focused on the development of cardiovascular disease risk among participants who were 18–30 years old. The study design, recruitment of participants, and methods have been described in detail elsewhere (Friedman et al. 1988). In brief, 5,115 African-American and white participants were recruited at baseline in 1985–1986 (year 0); follow-up examinations were completed at years 2, 5, 7, 10, 15, and 20 (2005–2006) for 91%, 86%, 81%, 79%, 74%, and 72%, respectively, of survivors. Institutional review board approval at each site and informed consent were obtained at every examination.

Prior to blood collection at year 2, participants were asked to fast for at least 12 hr. The serum samples that were collected and stored at this time (1987–1988) were used to measure POPs. Individuals were eligible for the current study if they had had no diagnosis of diabetes at years 0 and 2 and if they had been diagnosed with type 2 diabetes at any subsequent examination. Diabetes was defined as ever having taken antidiabetic medications or ever having had fasting glucose ≥ 126 mg/dL at two or more examinations. After 18 years of follow-up 116 persons had been diagnosed with type 2 diabetes; of these, 90 were randomly selected as cases. Control subjects were randomly selected by assigning a random number (distributed uniformly between 0 and 1) to each participant who had fasting glucose < 100 mg/dL at all five examinations (years 0, 7, 10, 15, and 20) when glucose was measured. We frequency matched the controls to cases using the following year 0 BMI strata: < 20, 20–24.9, 25–29.9, 30–39.9, and ≥ 40 kg/m^2^; within each stratum we selected those participants who had the lowest random number until the desired number of participants was reached.

We also used the National Health and Examination Survey (NHANES) 2003–2004 to compare serum concentrations of POPs between CARDIA subjects and current concentrations in the U.S. general population. NHANES is an ongoing survey designed to measure the health and nutritional status of the civilian noninstitutionalized U.S. population [Centers for Disease Control and Prevention (CDC) 2010].

### Measurements

Data on demographic variables, health behaviors, anthropometric data, and various clinical variables were measured at baseline and at follow-up examinations. Blood samples were collected after an overnight fast. BMI was derived from measured height and weight (kilograms per meter squared). Clinical variables included fasting glucose, triglycerides, and total cholesterol. The protocols for lipid and glucose determinations can be found elsewhere (Jacobs et al. 1999).

### POPs analyses

POPs were measured in serum samples (stored at Solomon Park Research Laboratories, Seattle, WA, USA) collected at year 2 (stored frozen at −70°C until analysis). The samples were analyzed by a previously published methodology (Sjodin et al. 2004) involving solid-phase extraction and final determination using gas chromatography isotope dilution high-resolution mass spectrometry (GC/ID-HRMS). The GC/ID-HRMS analysis was performed on a MAT95XP (ThermoFinnigan MAT, Bremen, Germany) instrument. The chromatographic separations were carried out on a 6890N gas chromatograph (Agilent Technologies, Atlanta, GA) at the CDC in Atlanta, GA, USA. Laboratory personnel were masked to all CARDIA data, including case–control status. A total of 55 POPs were measured: 9 OC pesticides, 35 PCB congeners, 10 polybrominated diphenyl ether (PBDE) congeners, and 1 polybrominated biphenyl (PBB) congener [see Supplemental Material, Table 1 (doi:10.1289/ehp.0901480)].

### Statistical methods

Lipid-standardized concentrations, the concentrations of serum POPs divided by total serum lipid content (milligrams per deciliter) calculated as 2.2 × serum cholesterol (milligrams per deciliter) + serum triglycerides (milligrams per deciliter) + 62.3 (Phillips et al. 2008), have generally been reported in epidemiological studies, because POPs are predominantly carried in the lipid component of the blood. In addition, lipid-standardized concentration has been regarded as better reflecting body burden than wet-weight concentrations (Lopez-Carrillo et al. 1999). However, if certain POPs disturb lipid metabolism as we previously observed (Lee et al. 2007b), wet-weight (i.e. unadjusted) concentrations should be examined because lipid concentrations may be intermediate in a causal chain linking POPs and type 2 diabetes (Howard 1999; Schisterman et al. 2005). Furthermore, according to a simulation study conducted by Schisterman et al. (2005), wet-weight concentrations adjusting for triglycerides and total cholesterol had less bias than did lipid-standardized concentrations. Thus, we present in this study wet-weight concentrations, and wet-weight concentrations adjusting for triglyceride and total cholesterol by including these two lipid profiles in the models as confounders. Findings using lipid-standardized concentrations are also provided in Supplemental Material, Tables 2 and 3 (doi:10.1289/ehp.0901480) for comparison.

We performed all analyses using SAS 9.1 (SAS Institute Inc., Cary, NC USA) to measure individual POPs and summary POPs. In the individual analyses, study subjects were categorized in quartiles of the distribution among controls of each POP. We included only measurements with more than 75% of values above the detection limit (31 POPs: 8 OC pesticides, 22 PCBs, and 1 PBB congeners) in the analyses; thus, subjects with nondetectable or low concentrations belonged to the lowest quartile of each POP.

We formed summary POPs measures by applying three rationales. The first summary measure combined all 31 POPs examined in the current study. The second measure was based on both prior cross-sectional findings (Lee et al. 2006b, 2007c) and the findings for individual POPs presented in this paper. For example, of the 12 POPs that were significantly associated with type 2 diabetes in previous cross-sectional studies, Lee et al. (2006b, 2007c) showed that 8 POPs (*trans*-nonachlor, oxychlordane, PCB74, PCB153, PCB170, PCB180, PCB187, and BB153) had the highest risk in the second quartile, although most failed to reach statistical significance in individual analyses. We hypothesized *a priori* that this low-dose effect was present because serum concentrations of the current study subjects were much higher than were those in previous cross-sectional studies (Lee et al. 2006b, 2007c). Among the remaining 19 POPs that were newly included in the current study, 8 POPs also showed similar low-dose effects. Considering these findings in individual analyses, we included in the second summary each POP that showed an odds ratio (OR) ≥ 1.5 in the second quartile in lipid-adjusted analyses in the present study. A summary that included POPs that showed OR ≥ 1.7 in any quartile yielded similar results and is therefore not shown. The third rationale for forming other summary measures was to use underlying biologic mechanisms that were suggested by others. For instance, McFarland and Clarke (1989) proposed PCB groupings by enzyme induction potential of cytochrome P450 subfamilies, whereas Wolff et al. (1997) classified them by estrogenic and neurotoxic, antiestrogenic (aryl hydrocarbon-receptor agonists, dioxin-like), and enzyme-inducing phenobarbital-type cytochrome P450 types.

In the summary analyses, we summed the individual ranks of several POPs. For example, in the case of POPs with no nondetectable values, the lowest concentration of a given POP was assigned rank 0, and the highest was assigned rank 179. For another particular POP for which 10 samples had values below LOD, subjects with concentrations below LOD were assigned rank 0, and the remaining 170 POPs were ranked by concentration, 10 for the lowest concentration and 179 for the highest concentration. The ranks for each POP were added, and summed values were divided into four categories (quartiles) or six categories (sextiles) in analyses that progressively traded sample size for lower POPs concentrations in the reference category.

ORs and 95% confidence intervals (CIs) for the risk of type 2 diabetes were estimated using logistic regression. The possible confounders included age (continuous), sex, race, and BMI (continuous) at year 2 in analyses of POPs wet weights; triglycerides (continuous) and total cholesterol (continuous) at year 2 were added in POPs lipid-adjusted analyses. The confounders were known risk factors of type 2 diabetes and also may be associated with serum concentrations of POPs. We present *p* for trend, *p* for quadratic term, or *p* for cubic term, representing each category by its median value (median values of serum concentrations for individual POPs measures and median ranks for summary POPs measures). The quadratic model allows the outcome curve to rise and then fall (as in an inverted U shape), whereas the cubic model would allow the outcome curve to rise, then to fall, then to rise again. We further examined if there were multiplicative interactions between individual sums of POPs (median ranks for sextiles) and obesity (< 30 vs. ≥ 30 kg/m^2^) on the risk of diabetes. Given the small sample size, we considered *p* < 0.05 as significant and *p* < 0.1 as borderline significant.

## Results

The average age of participants, both cases and controls, at year 2 was 27 years (Table 1). Cases had a higher proportion of men and a lower proportion of whites than did controls. Although the control distribution of BMI was frequency matched to the case distribution of BMI at year 0, mean BMI difference between cases and controls expanded by year 2. After BMI matching, we found significantly higher fasting triglycerides and glucose among cases than among controls, whereas high-density lipoprotein (HDL) cholesterol was correspondingly lower among cases. Low-density lipoprotein (LDL) cholesterol was not significantly different between cases and controls at baseline.

Most POPs concentrations, particularly OC pesticides, were much higher among the CARDIA participants assessed in 1987–1988 than among NHANES subjects assessed in 2003–2004 at 20–36 years old, which represents the age range of CARDIA participants at year 2 in 1987–1988. The POPs in CARDIA were also higher when compared with NHANES participants age 36–52 years (the age range that CARDIA participants had achieved in 2003–2004). Importantly, the second quartile POPs concentrations in the current population were similar to those in the highest quartile in the NHANES subjects. However, most PBDEs were not detectable in CARDIA subjects, whereas PBDEs were detectable in NHANES subjects, suggesting recent exposure to PBDEs (Table 2).

Tables 3–5 present two models for predicting incident diabetes from individual POPs and their summations: unadjusted lipid (model 1) and adjusted lipid (model 2). Most *p-*values for individual POPs failed to reach statistical significance. However, some individual POPs displayed statistically significantly higher ORs of diabetes in the second quartile compared with the first quartile, particularly before lipid adjustment. The results for individual POPs should be carefully interpreted with regard to effects of lipid adjustment, because POPs may promote dyslipidemia (so the lipid adjustment could be an overadjustment).

Among 8 OC pesticides, *trans*-nonachlor showed the strongest association with the risk of future diabetes (*p* for quadratic term = 0.06) (model 2 in the Table 3). Subjects in the second quartile had four times higher risk of developing type 2 diabetes compared with those in the lowest quartile. Oxychlordane and mirex also showed about two times higher risk in the second quartile, but these associations did not achieve statistical significance.

Among PCB congeners, highly chlorinated PCBs with seven or eight chlorides tended to show the highest risk in the second quartile with adjusted ORs of 2–3 (Table 4). Among moderately chlorinated PCBs, PCB74, PCB146, and PCB153 showed a high risk for type 2 diabetes in the second quartile. PBB153 also showed a higher risk for type 2 diabetes in the second or third quartiles (Table 4). On the other hand, we found marginally significant trends for PCB105, PCB194, PCB195, PCB196–203, and PCB209. However, the inverse trend was due to a decreased risk in third and fourth quartiles after adjusting for lipid profiles. PCB194, PCB195, and PCB196–203 still showed the highest risk in the second quartile despite the inverse trends.

**Figure 1:**
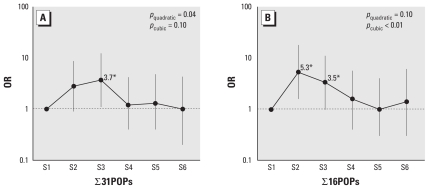
Adjusted ORs and 95% CIs of incident diabetes according to sextiles of the summary measure formed from serum concentrations of all 31 POPs (∑31POPs; *A*) or 16 selected POPs [∑16POPs; *B*) (*trans*-nonachlor + oxychlordane + mirex + PBB153 + 12 PCBs) with ORs ≥ 1.5 in the second quartile in Tables 3 and 4. Lipid-adjusted model, that is, adjusted for age, sex, race, BMI, triglycerides, and total cholesterol at year 2. *Significantly different from 1, *p* < 0.05.

We examined associations of the sum of POPs with incident type 2 diabetes. The summary measure with all 31 POPs showed an inverted U-shaped pattern, in particular in sextile analysis (Table 5; Figure 1). The increased risk in the last sextile disappeared after lipid adjustment. Because it is unlikely that all POPs would be equally involved in the association with incident diabetes, we further examined a second, more selective summary measure with POPs with ≥ 1.5 OR in the second quartile in the analyses of individual POPs and observed stronger associations at low serum concentrations than those of all 31 POPs (Table 5). When we used sextiles, the adjusted OR of the second sextile was 5.4 (95% CI, 1.6–18.4, model 2) and the risk decreased as the summary score increased, making an inverted U-shaped association. Because the highest risk was observed below the median summary score and because there was some tendency for risk to increase in the highest category, compared with next highest category in model 1 without lipid adjustment, *p* for cubic trend was more significant than *p* for quadratic trend in model 1. As was true for all 31 POPs, we found that the increased risk in the highest sextile disappeared after lipid adjustment.

We also examined several groupings of PCB congeners classified by biological characteristics (Table 5). Using McFarland and Clarke’s classification (1989), we found that phenobarbital-type inducers and weak or noninducers of CYP 450 showed low-dose effects, whereas mixed-type inducers did not show any association. Using the classification developed by Wolff et al. (1997), we found that PCBs belonging to group 1B (estrogenic, weak phenobarbital-type inducers, persistent) or group 2B (antiestrogenic and immunotoxic, dioxin-like, persistent) showed the strongest low-dose effects. Based on findings using this classification, the most notable common property of PCBs that was associated with type 2 diabetes was persistency of PCBs, rather than estrogenic or dioxin-like activity.

**Figure 2:**
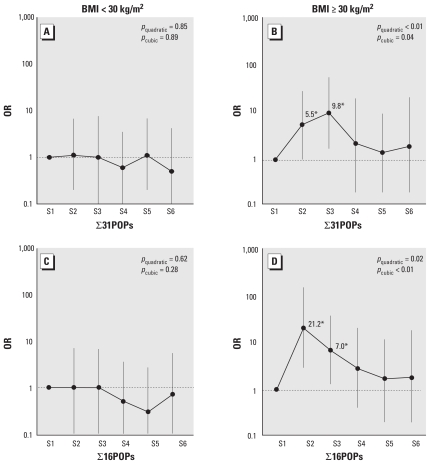
Adjusted ORs and 95% CIs of incident diabetes according to sextiles of the summary measure formed from serum concentrations of all 31 POPs (∑31POPs; *A, B*) or 16 selected POPs (∑16POPs; *C, D*) (*trans*-nonachlor + oxychlordane + mirex + PBB153 + 12 PCBs) with ORs ≥ 1.5 in the second quartile in Tables 3 and 4 stratified by year 2 BMI. Lipid-adjusted model, that is, adjusted for age, sex, race, BMI, triglycerides, and total cholesterol at year 2. *Significantly different from 1, *p* < 0.05.

When study subjects were stratified using a BMI cut point of 30 kg/m^2^, *p*-values for multiplicative scale interaction within logistic regression using product terms were marginally significant (*p* for interaction = 0.06) for the summary measure with 16 selected POPs with ≥ 1.5 OR in the second quartile. In accordance with our prior hypothesis, the inverted U-shaped association was evident only among subjects with BMI ≥ 30 kg/m^2^ for all 31 POPs and for 16 selected POPs (Figure 2). With the summary measure of 16 selected POPs with ≥ 1.5 ORs in the second quartile, compared with the lowest sextile, the risk in the second sextile was 21.2 times higher among those with BMI ≥ 30 kg/m^2^.

## Discussion

In this nested case–control study, we found that some POPs (in particular, *trans*-nonachlor and highly chlorinated PCBs) among the CARDIA participants at year 2 were associated with incident type 2 diabetes for the next 18 years, especially among obese persons. However, POPs did not show a traditional dose–response relation with diabetes. Instead POPs showed strong associations at relatively low exposure, making inverted U-shapes. These nonlinear shapes were anticipated, at least insofar as we discussed these possibilities in our previous cross-sectional studies on POPs (Lee et al. 2006a). In fact, the cohort study performed in Great Lakes Sport Fish Consumers, which included only 36 cases of incident diabetes, did not show the inverted U-shaped association in their main results (Turyk et al. 2009). However, low-dose effects were suggested among younger and heavier persons in their study, as in our study subjects (M Turyk, personal communication).

The inverted U-shaped associations have been proposed as possible biological responses of endocrine disruptors (Daston et al. 2003; Welshons et al. 2003), unlike the traditional paradigm of cellular toxicity in which there is a linear dose–response relation. POPs are well-known endocrine disruptors (Vasseur and Cossu-Leguille 2006). Hormones act indirectly through binding to specific receptors. In many biological systems, there is a linearity of dose and receptor occupancy only up to a dose that occupies about 10% of receptors. At higher doses, the effect of a higher occupancy rate does not increase linearly as the dose of the hormone increases (Daston et al. 2003; Welshons et al. 2003). Furthermore, a linear biological response is observed only at doses that result in the lowest range of receptor occupancy (Daston et al. 2003; Welshons et al. 2003). In high-dose hormone exposure, there is even downregulation of receptors as the dose further increases (Medlock et al. 1991). Thus, high doses of hormonally active chemicals can exert inhibitory effects on processes that are stimulated at much lower doses, which result in inverted U dose–response curves. Although endocrine disruption is an example of a biological system that could lead to low-dose response, we do not have specific evidence that endocrine disruption explains these epidemiologic observations.

At present, there is little knowledge about the biological mechanisms that might link POPs and type 2 diabetes. Although aryl hydrocarbon receptor (AhR) affinity has been the main focus in relation to POPs toxicity (Nebert et al. 1993), PCBs with some affinity to AhR (PCB105, PCB118, and PCB156) were not clearly associated with the risk of type 2 diabetes. Thus, mechanisms other than AhR affinity may be involved in the pathogenesis of type 2 diabetes. In addition, when we analyzed PCBs using the classification of Wolff et al. (1997), we found that groupings of both estrogenic PCBs and antiestrogenic PCBs showed low-dose effects. Thus, estrogenicity, at least in the Wolff et al. (1997) formulation, does not explain the link between PCBs and diabetes. In the analyses, using the classification of McFarland and Clarke, (1989), we found that PCBs in a grouping of phenobarbital-type inducers and in another grouping of weak or noninducers of CYP 450 showed the low-dose effects, whereas groupings of PCBs that were mixed-type inducers were not associated with type 2 diabetes. Interestingly, PCBs in the grouping of mixed-type inducers have been traditionally regarded as having the greatest toxic potential compared with other PCBs. Therefore, our data suggest that neither an AhR affinity nor estrogenicity explain the association between PCBs and type 2 diabetes. Rather, chronic exposure with weak or little-known toxicity may play a role in the pathogenesis of type 2 diabetes, given that highly chlorinated PCBs tend to be more persistent in the environment than PCBs with fewer chlorines. In the absence of known biological mechanism(s) and demonstrated low-dose effects of POPs on type 2 diabetes, our grouping of individual POP analyses, coupled with the consistency of our results with previous findings for prevalent diabetes (Lee et al. 2006b, 2007c), can be justified to create summary POPs measures based on results of individual POP analyses.

For valid estimation of relative risks to detect the presence of low-dose effects, a reference group is required with extremely low concentrations of relevant POPs so that low risk is uniform across values within the reference group. However, a true reference group without any exposure to POPs does not exist in the general population (i.e., in epidemiological data). In general, serum concentrations of POPs tend to be highly correlated with each other because of the simultaneous exposure to various POPs through food consumption, but the correlations are not perfect. Given these correlations, the reference group used in the analysis of an individual POP would have the lowest concentration of that specific POP but could have higher concentrations of other POPs that had a higher risk of type 2 diabetes. Therefore, in the presence of low-dose effects of POPs, the contamination of the reference group of specific POPs with higher concentrations of other important POPs may lead to an artificial increase of risk within the reference group of the specific POP, leading to underestimated risk relative to this group. In this situation, a summary POPs measures formed by summing individual ranks of the POPs, for example, those 16 POPs with ORs of ≥ 1.5 in the second quartile in individual analyses, facilitates selecting a reference group with extremely low concentrations of those POPs that are relevant to a risk of type 2 diabetes. We acknowledge that such a summary measure may to some extent overestimate ability to predict type 2 diabetes because it is data driven; however, this tendency is countered by the fact that several of the 16 POPs selected for analysis in this study were based on *a priori* selection of POPs that were found to be important in previous studies (Lee et al. 2006b, 2007c).

In contrast, although the summary measure of all 31 POPs is not data driven, it can compromise our effort to select subjects with very low concentrations of relevant POPs as the reference group. For example, some subjects in the lowest sextile of the summary measure of all 31 POPs were those with very low concentrations of the irrelevant POPs but relatively high concentrations of the 16 POPs with ORs of ≥ 1.5 in the second quartile. In this case, the risk of diabetes in the reference group, based on the sum of all 31 POPs, would increase and the OR estimates would be biased toward the null, compared with using the reference group based on the sum of the 16 selected POPs. The true association of selected POPs with diabetes risk likely lies between the estimates based on these two summary measures. Whichever summary measure we used, the ORs detected were large, particularly among the obese participants, so the findings as presented give a good idea of the size and shape of the association of certain POPs with incident diabetes.

In this study, another important strategy to reduce POPs level in the reference group was to categorize in sextiles. This strategy could have the drawback of leading to too many categories in a study with a relatively small sample size. In fact, our study showed that the associations became stronger when we refined the lowest category by expanding from quartiles to sextiles.

One critical question about exposure assessment is whether lipid standardization of POPs is valid in epidemiological studies. In a previous study, Lee et al. (2006a) reported that OC pesticides and PCBs were associated with dyslipidemia; that is, increased triglycerides and decreased HDL cholesterol. Our findings in this study also suggest that some POPs may disturb lipid metabolism over the extended period of observation; these findings will be reported in a separate paper. As dyslipidemia is an early manifestation of conditions characterized by insulin resistance and is often detectable before the development of postprandial or fasting hyperglycemia (Lewis et al. 2002), lipid concentrations may be intermediaries in the associations between POPs and type 2 diabetes. In this case, any form of adjustment for circulating lipid concentrations would substantially underestimate true risk associations. However, although it can be overadjustment to control for baseline lipid concentrations, findings not adjusted for lipids can be underadjusted, because POPs are carried by lipid in the blood. In fact, the effect of lipid adjustment was most strongly observed in the highest sextile of POPs, which consisted of subjects with a broad range of particularly high lipid concentrations. The highest category tended toward increased ORs before lipid adjustment, but this tendency mostly disappeared after lipid adjustment. The true associations between the POPs studied and incident diabetes may lie between the estimates with and without lipid adjustment.

Another important methodological issue is that a single exposure assessment provides an imprecise picture of POPs exposure. In this study, serum was sampled up to 18 years before the diabetes diagnosis. Although POPs have long half-lives, body burden of POPs can change substantially during follow-up. Because the development of diabetes requires deterioration of glucose metabolism over the long term, prolonged and persistent exposure to POPs may be an important factor in a putative causal pathway linking POPs and diabetes. Because we measured POPs only once, we could not assess whether their concentrations changed during follow-up. In fact, chlorinated POP concentrations at year 2 in these CARDIA subjects (1987–1988, recognizing that they had higher BMI than the general CARDIA sample) were much higher than those in the current U.S general population—a finding that suggests that chlorinated POPs concentrations in serum of CARDIA subjects primarily reflect the body burden accumulated during the calendar time before banning chlorinated POPs in the 1970s. Based on the comparison with NHANES participants at current CARDIA ages, current concentrations of chlorinated POPs, not brominated POPs such as PBDEs, in our participants would be lower than at year 2. However, we also believe that the slow clearances of POPs over many years differ among people, and we hypothesize that the rank of POPs concentrations of the participants may have changed during follow-up. It is thought that there are interindividual and chemical-specific variations in excretion of POPs (Thomaseth and Salvan 1998; Wolff et al. 1997).

As most chlorinated POPs have already been banned and body burdens are decreasing, research interest has tended to focus on more recently introduced POPs such as PBDEs. However, the strong low-dose effects of chlorinated POPs observed here imply that their low persistent exposures throughout the body may be a problem well into the future as the banned POPs slowly clear, passing through perhaps more dangerous lower concentrations. In addition, obesity seemed to exaggerate metabolic disturbance related to POPs, with the result that in the midst of the ongoing obesity epidemic, the currently low POPs concentrations may be a potent health risk.

This study has several limitations. First, the sample size was a compromise related to the practicalities of cost and the use of precious samples. Therefore, most analyses based on individual POPs failed to reach statistical significance in terms of *p* for trend or *p* for quadratic term, although some ORs in the second quartile showed statistical significance. Second, the data analyses involved a large number of statistical tests; therefore, some of the individual statistically significant findings may have occurred by chance. In this study, we focused our interpretation more on consistency than on statistical significance. Third, under the low-dose effect, the selection of a reference group with very close to zero POP concentrations is critical to unbiased assessment of the risk of adverse health outcomes. Combining study subjects across apparently low ranges of POPs can lead to a reference group with artificially increased risk and diluted relative risk estimates. As the CARDIA subjects in 1987–1988 had higher values of chlorinated POPs than present in the NHANES studies in 2003–2004, we cannot exclude the possibility that some risk gradient was still masked in our CARDIA subgroup, even when we used the lowest control group sextile to approximate more closely a true zero risk reference group in our nested case–control study. Finally, we could not exclude the possibility that other POPs not measured in this study could play a role in the pathogenesis of type 2 diabetes, because serum concentrations of POPs are highly correlated in the general population.

In conclusion, the results from this study suggest that environmental exposure to some POPs may increase, in a nonlinear fashion, the risk of future type 2 diabetes in the general population. Various POPs, which mainly accumulate in adipose tissue, may play a critical role in the current epidemic of type 2 diabetes.

## Figures and Tables

**Table 1:** Baseline characteristics of CARDIA subjects.

Characteristic	Control (*n* = 90)	Case (*n* = 90)	*p*-Value
Age at year 2 (years)	27.2 ± 3.3	27.2 ± 3.6	0.91
Percent men	31.1	45.6	0.05
Percent white	51.1	31.1	< 0.01
BMI (kg/m^2^)			
Year 0	28.4 ± 6.1	29.6 ± 6.4	0.19
Year 2	29.1 ± 6.7	30.9 ± 6.6	0.07
Triglycerides (mg/dL)			
Year 0	74.1 ± 46.7	94.6 ± 65.1	0.02
Year 2	78.4 ± 46.4	110.2 ± 70.9	< 0.01
HDL cholesterol (mg/dL)			
Year 0	53.2 ± 13.5	47.5 ± 12.9	< 0.01
Year 2	53.2 ± 13.7	45.7 ± 12.2	< 0.01
LDL cholesterol (mg/dL)			
Year 0	113.1 ± 21.8	116.2 ± 32.0	0.52
Year 2	111.5 ± 32.9	118.6 ± 32.1	0.15
Fasting glucose (mg/dL)			
Year 0	79.8 ± 6.0	86.1 ± 10.6	< 0.01

Abbreviations: HDL, high-density lipoprotein; LDL, low-density lipoprotein. Tabulated values are mean ± SD.

**Table 2:** Comparison of serum concentrations (pg/g) of several selected POPs between CARDIA control^a^ and NHANES^b^ participants.

Data set	Age range (years)	Detectable rate (%)	Quartiles			
			1	2	3	4
Oxychlordane						
CARDIA 1987–1988	20–36	99.4	≤ 110	111–157	158–200	> 200
NHANES 2003–2004	20–36	72.6	—	≤ 30	31–52	> 52
NHANES 2003–2004	36–52	92.1	≤ 46	47–70	71–108	> 108

*trans*-Nonachlor						
CARDIA 1987–1988	20–36	100	≤ 109	110–174	175–250	> 250
NHANES 2003–2004	20–36	98.6	≤ 27	28–51	52–88	> 88
NHANES 2003–2004	36–52	98.5	≤ 61	62–101	102–169	> 169

β-Hexachlorocyclohexane						
CARDIA 1987–1988	20–36	98.3	≤ 75	76–108	109–146	> 146
NHANES 2003–2004	20–36	54.4	—	≤ 19	20–34	> 34
NHANES 2003–2004	36–52	79.7	≤ 22	23–43	44–70	> 71

*p,p′*--DDE						
CARDIA 1987–1988	20–36	100	≤ 2,153	2,154–3,312	3,313–5,731	> 5,731
NHANES 2003–2004	20–36	99.6	≤ 462	463–759	760–1,275	> 1,275
NHANES 2003–2004	36–52	100	≤ 846	847–1,444	1,445–3,035	> 3,035

Mirex						
CARDIA 1987–1988	20–36	75	—	≤ 21	22–34	> 34
NHANES 2003–2004	20–36	15.1	—	—	—	—
NHANES 2003–2004	36–52	51.7	—	—	≤ 32	> 32

PCB74						
CARDIA 1987–1988	20–36	100	≤ 58	59–98	99–144	> 144
NHANES 2003–2004	20–36	100	≤ 11	12–15	16–22	> 22
NHANES 2003–2004	36–52	100	≤ 22	23–31	32–48	> 48

PCB153						
CARDIA 1987–1988	20–36	100	≤ 204	205–349	350–466	> 466
NHANES 2003–2004	20–36	100	≤ 37	38–55	56–89	> 89
NHANES 2003–2004	36–52	100	≤ 106	107–155	156–231	> 231

PCB178						
CARDIA 1987–1988	20–36	95.6	≤ 8	9–15	16–21	> 21
NHANES 2003–2004	20–36	74.4	≤ 1	2–3	4–5	> 5
NHANES 2003–2004	36–52	97.7	≤ 6	7–9	10–13	> 13

PCB187						
CARDIA 1987–1988	20–36	100	≤ 44	45–78	79–104	> 104
NHANES 2003–2004	20–36	98.4	≤ 6	7–11	12–19	> 19
NHANES 2003–2004	36–52	97.2	≤ 23	24–35	36–54	> 54

PBB153						
CARDIA 1987–1988	20–36	100	≤ 9	10–16	17–23	> 23
NHANES 2003–2004	20–36	78.0	≤ 3	4–9	10–17	> 17
NHANES 2003–2004	36–52	88.7	≤ 10	11–18	19–34	> 34

—, nondetectable.

a CARDIA control subjects (*n* = 90) were categorized by quartile.

b Percentile values in NHANES were computed to account for the NHANES complex survey design. Dashes indicate undetectable concentrations.

**Table 3:** Adjusted ORs of incident diabetes according to quartiles of OC pesticides.

	Quartiles			
	1	2	3	4
Oxychlordane				
Case–control	13/22	30/23	16/22	31/23
Model 1	Reference	2.0 (0.8–5.0)	1.3 (0.4–3.7)	2.6 (1.0–7.0)
Model 2	Reference	1.7 (0.7–4.3)	1.1 (0.4–3.4)	1.4 (0.5–4.4)

*trans*-Nonachlor				
Case–control	7/22	33/23	22/22	28/23
Model 1	Reference	4.8 (1.7–13.7)	2.7 (0.9–8.3)	3.7 (1.2–11.0)
Model 2	Reference	4.3 (1.5–12.6)	2.3 (0.7–7.4)	2.0 (0.6–6.9)

Hexachlorobenzene				
Case–control	25/22	18/23	21/22	26/23
Model 1	Reference	0.7 (0.3–1.7)	0.9 (0.4–2.3)	1.4 (0.6–3.5)
Model 2	Reference	0.5 (0.2–1.3)	0.7 (0.3–1.9)	1.0 (0.4–2.6)

β-Hexachlorocyclohexane				
Case–control	22/22	23/23	19/22	26/23
Model 1	Reference	1.2 (0.5–3.0)	1.2 (0.5–3.2)	1.4 (0.5–3.4)
Model 2	Reference	1.0 (0.4–2.5)	0.9 (0.3–2.5)	0.8 (0.3–2.2)

γ-Hexachlorocyclohexane				
Case–control	19/22	21/23	24/22	26/23
Model 1	Reference	1.2 (0.5–3.0)	1.2 (0.5–3.0)	1.3 (0.5–3.1)
Model 2	Reference	1.2 (0.5–3.0)	1.1 (0.4–2.8)	1.3 (0.5–3.2)

*p,p′*-DDE				
Case–control	18/22	23/23	30/22	19/23
Model 1	Reference	1.3 (0.5–3.3)	1.6 (0.7–3.9)	0.9 (0.3–2.4)
Model 2	Reference	1.2 (0.5–3.0)	1.3 (0.5–3.4)	0.7 (0.2–1.9)

*p,p′*-DDT				
Case–control	17/22	16/23	25/22	32/23
Model 1	Reference	0.9 (0.3–2.2)	1.3 (0.5–3.3)	1.3 (0.5–3.3)
Model 2	Reference	0.8 (0.3–2.2)	1.0 (0.4–2.5)	0.9 (0.3–2.6)

Mirex				
Case–control	16/29	18/16	21/22	35/23
Model 1	Reference	2.1 (0.8–5.5)	1.5 (0.6–3.7)	2.1 (0.8–5.1)
Model 2	Reference	2.0 (0.7–5.5)	1.4 (0.5–3.7)	1.8 (0.7–4.9)

Model 1: adjusted for age, sex, race, and BMI at year 2. Model 2: further adjustment for triglyceride and total cholesterol at year 2.

**Table 4:** Adjusted ORs of incident diabetes according to quartiles of PCB or PBB.

Congener (no. of chlorine or bromine atoms)	Quartiles			
	1	2	3	4
PCB74 (4)				
Case–control	17/22	33/23	16/22	24/23
Model 1	Reference	2.8 (1.1–7.5)	1.4 (0.5–4.0)	2.6 (0.9–7.1)
Model 2	Reference	2.8 (1.0–7.3)	0.9 (0.3–2.6)	1.4 (0.5–4.5)

PCB87 (5)				
Case–control	22/22	18/22	25/23	25/23
Model 1	Reference	1.0 (0.4–2.5)	1.3 (0.5–3.1)	1.0 (0.4–2.4)
Model 2	Reference	1.1 (0.4–2.8)	1.1 (0.5–2.9)	0.6 (0.2–1.6)

PCB99 (5)				
Case–control	21/22	18/23	22/22	29/23
Model 1	Reference	0.8 (0.3–2.0)	1.1 (0.5–2.8)	1.3 (0.5–3.2)
Model 2	Reference	0.7 (0.3–1.8)	0.8 (0.3–2.1)	0.8 (0.3–2.0)

PCB105 (5)				
Case–control	28/21	18/24	22/22	22/23
Model 1	Reference	0.5 (0.2–1.2)	0.8 (0.3–2.1)	0.6 (0.2–1.6)
Model 2	Reference	0.3 (0.1–0.8)	0.5 (0.2–1.4)	0.2 (0.1–0.8)

PCB118 (5)				
Case–control	26/22	18/23	24/22	22/23
Model 1	Reference	0.6 (0.2–1.6)	1.1 (0.5–2.8)	0.8 (0.3–2.1)
Model 2	Reference	0.5 (0.2–1.3)	0.7 (0.3–1.8)	0.5 (0.2–1.4)

PCB146 (6)				
Case–control	14/22	32/22	17/23	27/23
Model 1	Reference	2.2 (0.9–5.7)	1.1 (0.4–3.1)	1.7 (0.6–4.6)
Model 2	Reference	1.7 (0.7–4.5)	0.8 (0.3–2.4)	0.9 (0.3–2.6)

PCB153 (6)				
Case–control	15/22	35/23	14/22	24/23
Model 1	Reference	2.4 (1.0–6.1)	0.8 (0.3–2.5)	1.6 (0.6–4.4)
Model 2	Reference	2.0 (0.8–5.2)	0.6 (0.2–1.9)	0.8 (0.2–2.6)

PCB156 (6)				
Case–control	17/21	29/24	20/22	24/23
Model 1	Reference	1.7 (0.7–4.3)	1.1 (0.4–3.1)	1.6 (0.6–4.7)
Model 2	Reference	1.3 (0.5–3.5)	0.9 (0.3–2.6)	0.8 (0.2–2.9)

PCB157 (6)				
Case–control	20/21	29/23	21/23	20/23
Model 1	Reference	1.2 (0.5–3.1)	0.7 (0.3–1.9)	1.0 (0.3–2.9)
Model 2	Reference	1.0 (0.4–2.5)	0.5 (0.2–1.5)	0.5 (0.1–1.7)

PCB138–158 (6)				
Case–control	20/23	26/22	17/22	27/23
Model 1	Reference	1.4 (0.6–3.5)	0.8 (0.3–2.2)	1.4 (0.5–3.4)
Model 2	Reference	1.2 (0.5–2.9)	0.6 (0.2–1.6)	0.8 (0.3–2.2)

PCB167 (6)				
Case–control	24/22	23/23	25/22	18/23
Model 1	Reference	1.1 (0.5–2.6)	1.3 (0.5–3.1)	0.9 (0.3–2.2)
Model 2	Reference	0.9 (0.4–2.2)	1.0 (0.4–2.5)	0.5 (0.2–1.3)

PCB170 (7)				
Case–control	13/22	35/23	18/22	24/23
Model 1	Reference	2.7 (1.0–7.1)	1.1 (0.4–3.3)	1.9 (0.6–5.9)
Model 2	Reference	2.2 (0.8–6.1)	0.8 (0.2–2.6)	0.9 (0.3–3.4)

PCB178 (7)				
Case–control	14/22	38/23	11/21	27/24
Model 1	Reference	3.2 (1.3–8.2)	0.7 (0.2–2.2)	2.0 (0.7–5.5)
Model 2	Reference	2.7 (1.0–7.0)	0.5 (0.2–1.7)	1.0 (0.3–3.2)

PCB180 (7)				
Case–control	12/22	41/23	13/22	24/23
Model 1	Reference	3.4 (1.3–9.0)	0.9 (0.3–2.8)	2.0 (0.6–6.4)
Model 2	Reference	2.8 (1.0–7.6)	0.7 (0.2–2.5)	1.1 (0.3–3.9)

PCB183 (7)				
Case–control	15/22	31/23	18/22	26/23
Model 1	Reference	1.9 (0.8–4.8)	1.3 (0.5–3.4)	1.5 (0.6–3.9)
Model 2	Reference	1.7 (0.7–4.2)	1.0 (0.3–2.7)	0.8 (0.3–2.3)

PCB187 (7)				
Case–control	12/22	41/23	8/22	29/23
Model 1	Reference	3.2 (1.2–8.2)	0.5 (0.2–1.8)	1.9 (0.7–5.4)
Model 2	Reference	2.8 (1.1–7.4)	0.5 (0.1–1.6)	1.0 (0.3–3.3)

PCB194 (8)				
Case–control	16/22	32/23	23/22	19/23
Model 1	Reference	2.0 (0.8–5.1)	1.2 (0.4–3.3)	1.1 (0.3–3.4)
Model 2	Reference	1.6 (0.6–4.3)	0.8 (0.2–2.4)	0.4 (0.1–1.5)

PCB195 (8)				
Case–control	15/22	34/22	17/23	24/23
Model 1	Reference	2.3 (0.9–5.9)	1.0 (0.4–2.9)	1.3 (0.5–3.8)
Model 2	Reference	1.9 (0.7–5.1)	0.8 (0.3–2.2)	0.6 (0.2–1.9)

PCB199 (8)				
Case–control	16/22	35/23	14/22	25/23
Model 1	Reference	2.3 (0.9–5.8)	0.9 (0.3–2.5)	1.6 (0.5–4.6)
Model 2	Reference	2.2 (0.8–5.6)	0.7 (0.2–2.3)	0.9 (0.3–3.0)

PCB196–203 (8)				
Case–control	15/22	39/23	13/22	23/23
Model 1	Reference	2.8 (1.1–7.0)	0.8 (0.3–2.4)	1.5 (0.5–4.3)
Model 2	Reference	2.3 (0.9–6.1)	0.6 (0.2–1.9)	0.6 (0.2–2.2)

PCB206 (9)				
Case–control	20/22	19/23	29/22	22/23
Model 1	Reference	0.9 (0.3–2.2)	1.3 (0.5–3.4)	0.9 (0.3–2.5)
Model 2	Reference	0.7 (0.3–1.9)	1.0 (0.4–2.7)	0.5 (0.1–1.6)

PCB209 (10)				
Case–control	16/19	35/26	15/22	24/23
Model 1	Reference	1.6 (0.6–4.1)	0.6 (0.2–1.8)	0.9 (0.3–2.7)
Model 2	Reference	1.1 (0.4–3.0)	0.3 (0.1–1.1)	0.4 (0.1–1.3)

PBB153 (6)				
Case–control	10/22	29/23	26/22	25/23
Model 1	Reference	3.0 (1.1–8.1)	3.1 (1.1–9.2)	2.6 (0.9–7.9)
Model 2	Reference	2.5 (0.9–6.9)	2.5 (0.8–7.6)	1.8 (0.6–5.8)

Model 1: adjusted for age, sex, race, and BMI at year 2. Model 2: further adjustment for triglyceride and total cholesterol at year 2.

**Table 5:** Adjusted ORs of incident diabetes according to quartiles or sextiles of summary measures of POPs.

Quartile/sextile	1	2	3	4	5	6	*p*_quadratic_	*p*_cubic_
All 31 POPs in Tables 3 and 4								
Quartile								
Model 1	Reference	1.9 (0.8–4.5)	1.0 (0.4–2.5)	1.2 (0.5–3.2)			0.47	0.14
Model 2	Reference	1.4 (0.5–3.5)	0.7 (0.2–1.9)	0.5 (0.1–1.5)			0.22	0.33
Sextile								
Model 1	Reference	2.9 (1.0–8.8)	4.8 (1.5–14.8)	1.5 (0.4–4.8)	1.9 (0.6–4.8)	2.7 (0.8–8.8)	0.20	0.03
Model 2	Reference	2.8 (0.9–8.7)	3.7 (1.1–12.3)	1.2 (0.4–4.2)	1.3 (0.4–4.8)	1.0 (0.2–4.3)	0.04	0.10

16 POPs with OR ≥ 1.5 in the second quartile in Tables 3 and 4								
*trans*-Nonachlor, oxychlordane, mirex, PCB74, PCB146, PCB153, PCB170, PCB178, PCB180, PCB183, PCB187, PCB194, PCB195, PCB199, PCB196–203, PBB 153								
Quartile								
Model 1	Reference	3.4 (1.4–8.7)	1.1 (0.4–3.0)	1.6 (0.6–4.3)			0.14	0.01
Model 2	Reference	2.8 (1.1–7.2)	0.8 (0.3–2.3)	0.7 (0.2–2.2)			0.05	0.04
Sextile								
Model 1	Reference	5.9 (1.8–19.3)	4.3 (1.3–13.9)	2.0 (0.6–7.0)	1.4 (0.4–5.2)	3.9 (1.1–13.9)	0.41	< 0.01
Model 2	Reference	5.4 (1.6–18.4)	3.4 (1.0–11.4)	1.6 (0.4–5.8)	1.0 (0.3–4.1)	1.4 (0.3–6.3)	0.10	< 0.01

McFarland and Clark (1989) groupings								
Group 1B (mixed-type inducers: PCB105, PCB118, PCB156, PCB170)								
Sextile								
Model 1	Reference	1.0 (0.3–2.9)	1.0 (0.3–2.9)	2.1 (0.7–6.5)	1.0 (0.3–3.0)	1.4 (0.4–4.2)	0.76	0.91
Model 2	Reference	0.9 (0.3–2.8)	0.8 (0.3–2.6)	1.4 (0.4–4.6)	0.5 (0.2–1.9)	0.7 (0.2–2.5)	0.58	0.90

Group 2 (phenobarbital-type inducers: PCB87, PCB99, PCB153, PCB180, PCB183, PCB194)								
Sextile								
Model 1	Reference	2.1 (0.7–6.4)	2.5 (0.8–7.5)	1.0 (0.3–3.2)	1.2 (0.4–3.8)	1.6 (0.5–5.0)	0.43	0.08
Model 2	Reference	2.0 (0.7–6.4)	2.0 (0.6–6.2)	0.9 (0.3–3.0)	0.7 (0.2–2.5)	0.6 (0.2–2.4)	0.12	0.17

Group 3 (weak or noninducers: PCB74, PCB187)								
Sextile								
Model 1	Reference	1.1 (0.4–3.4)	3.7 (1.2–11.4)	1.9 (0.6–5.8)	1.3 (0.4–4.3)	2.0 (0.6–6.3)	0.21	0.34
Model 2	Reference	1.0 (0.3–3.2)	2.9 (0.9–9.3)	1.4 (0.4–4.8)	0.9 (0.3–3.0)	0.9 (0.2–3.5)	0.12	0.50

Wolff et al. (1997) groupings								
Group 1B (estrogenic, weak phenobarbital-type inducers, persistent: PCB187)								
Sextile								
Model 1	Reference	2.8 (0.8–8.5)	3.2 (1.0–9.7)	1.0 (0.3–3.2)	1.2 (0.4–3.9)	2.7 (0.8–8.9)	0.79	< 0.01
Model 2	Reference	2.4 (0.8–7.9)	2.8 (0.8–8.9)	0.8 (0.2–2.7)	0.8 (0.2–3.0)	1.1 (0.3–4.4)	0.35	0.02

Group 2A (antiestrogenic, immunotoxic, dioxin-like, moderately persistent: PCB74, PCB105, PCB118, PCB156, PCB167)								
Sextile								
Model 1	Reference	0.9 (0.3–2.5)	0.7 (0.2–2.1)	1.6 (0.5–5.0)	0.8 (0.2–2.4)	0.9 (0.3–2.9)	0.93	0.59
Model 2	Reference	0.8 (0.3–2.5)	0.5 (0.2–1.7)	0.9 (0.3–3.1)	0.4 (0.1–1.3)	0.5 (0.1–1.8)	0.93	0.96

Group 2B (antiestrogenic and immunotoxic, dioxin-like, persistent: PCB170)								
Sextile								
Model 1	Reference	3.2 (1.0–10.1)	3.1 (1.0–9.7)	1.3 (0.4–4.2)	1.2 (0.3–4.3)	2.9 (0.8–10.3)	0.65	< 0.01
Model 2	Reference	2.8 (0.9–9.1)	2.5 (0.8–8.3)	0.9 (0.3–3.4)	0.9 (0.2–3.4)	1.2 (0.3–5.2)	0.31	0.02

Group 3 (phenobarbital-type inducers, persistent: PCB99, PCB153, PCB180, PCB183)								
Sextile								
Model 1	Reference	1.6 (0.6–4.9)	1.8 (0.6–5.4)	0.9 (0.3–2.7)	1.1 (0.3–3.5)	1.8 (0.6–5.9)	0.93	0.12
Model 2	Reference	1.5 (0.5–4.6)	1.5 (0.5–4.6)	0.7 (0.2–2.3)	0.8 (0.2–2.7)	0.8 (0.2–3.1)	0.64	0.26

Model 1: wet-weight model, adjusted for age, sex, race, and BMI at year 2. Model 2: lipid-adjusted model 1 with further adjustment for triglycerides and total cholesterol at year 2.

## References

[b1-ehp-118-1235] Centers for Disease Control and Prevention (CDC) (2010). National Health and Nutrition Examination Survey: NHANES 2003–2004.

[b2-ehp-118-1235] Daston GP, Cook JC, Kavlock RJ (2003). Uncertainties for endocrine disrupters: our view on progress. Toxicol Sci.

[b3-ehp-118-1235] Friedman GD, Cutter GR, Donahue RP, Hughes GH, Hulley SB, Jacobs DR (1988). CARDIA: study design, recruitment, and some characteristics of the examined subjects. J Clin Epidemiol.

[b4-ehp-118-1235] Henriksen GL, Ketchum NS, Michalek JE, Swaby JA (1997). Serum dioxin and diabetes mellitus in veterans of Operation Ranch Hand. Epidemiology.

[b5-ehp-118-1235] Howard BV (1999). Insulin resistance and lipid metabolism. Am J Cardiol.

[b6-ehp-118-1235] Jacobs DR, Hannan PJ, Wallace D, Liu K, Williams OD, Lewis CE (1999). Interpreting age, period and cohort effects in plasma lipids and serum insulin using repeated measures regression analysis: the CARDIA Study. Stat Med.

[b7-ehp-118-1235] Lee DH, Jacobs DR, Porta M (2006a). Could low-level background exposure to persistent organic pollutants contribute to the social burden of type 2 diabetes?. J Epidemiol Community Health.

[b8-ehp-118-1235] Lee DH, Lee IK, Jin SH, Steffes M, Jacobs DR (2007a). Association between serum concentrations of persistent organic pollutants and insulin resistance among nondiabetic adults: results from the National Health and Nutrition Examination Survey 1999–2002. Diabetes Care.

[b9-ehp-118-1235] Lee DH, Lee IK, Porta M, Steffes M, Jacobs DR (2007b). Relationship between serum concentrations of persistent organic pollutants and the prevalence of metabolic syndrome among non-diabetic adults: results from the National Health and Nutrition Examination Survey 1999–2002. Diabetologia.

[b10-ehp-118-1235] Lee DH, Lee IK, Song K, Steffes M, Toscano W, Baker BA (2006b). A strong dose-response relation between serum concentrations of persistent organic pollutants and diabetes: results from the National Health and Examination Survey 1999–2002. Diabetes Care.

[b11-ehp-118-1235] Lee DH, Lee IK, Steffes M, Jacobs DR (2007c). Extended analyses of the association between serum concentrations of persistent organic pollutants and diabetes. Diabetes Care.

[b12-ehp-118-1235] Lewis GF, Carpentier A, Adeli K, Giacca A (2002). Disordered fat storage and mobilization in the pathogenesis of insulin resistance and type 2 diabetes. Endocr Rev.

[b13-ehp-118-1235] Li QQ, Loganath A, Chong YS, Tan J, Obbard JP (2006). Persistent organic pollutants and adverse health effects in humans. J Toxicol Environ Health A.

[b14-ehp-118-1235] Longnecker MP, Michalek JE (2000). Serum dioxin level in relation to diabetes mellitus among Air Force veterans with background levels of exposure. Epidemiology.

[b15-ehp-118-1235] Lopez-Carrillo L, Torres-Sanchez L, Lopez-Cervantes M, Blair A, Cebrian ME, Uribe M (1999). The adipose tissue to serum dichlorodiphenyldichloroethane (DDE) ratio: some methodological considerations. Environ Res.

[b16-ehp-118-1235] McFarland VA, Clarke JU (1989). Environmental occurrence, abundance, and potential toxicity of polychlorinated biphenyl congeners: considerations for a congener-specific analysis. Environ Health Perspect.

[b17-ehp-118-1235] Medlock KL, Lyttle CR, Kelepouris N, Newman ED, Sheehan DM (1991). Estradiol down-regulation of the rat uterine estrogen receptor. Proc Soc Exp Biol Med.

[b18-ehp-118-1235] Nebert DW, Puga A, Vasiliou V (1993). Role of the Ah receptor and the dioxin-inducible [Ah] gene battery in toxicity, cancer, and signal transduction. Ann NY Acad Sci.

[b19-ehp-118-1235] Needham LL, Barr DB, Caudill SP, Pirkle JL, Turner WE, Osterloh J (2005). Concentrations of environmental chemicals associated with neurodevelopmental effects in U.S. population. Neurotoxicology.

[b20-ehp-118-1235] Phillips KP, Foster WG, Leiss W, Sahni V, Karyakina N, Turner MC (2008). Assessing and managing risks arising from exposure to endocrine-active chemicals. J Toxicol Environ Health B Crit Rev.

[b21-ehp-118-1235] Schisterman EF, Whitcomb BW, Louis GM, Louis TA (2005). Lipid adjustment in the analysis of environmental contaminants and human health risks. Environ Health Perspect.

[b22-ehp-118-1235] Sjödin A, Jones RS, Lapeza CR, Focant JF, McGahee EE, Patterson DG (2004). Semiautomated high-throughput extraction and cleanup method for the measurement of polybrominated diphenyl ethers, polybrominated biphenyls, and polychlorinated biphenyls in human serum. Anal Chem.

[b23-ehp-118-1235] Steenland K, Piacitelli L, Deddens J, Fingerhut M, Chang LI (1999). Cancer, heart disease, and diabetes in workers exposed to 2,3,7,8-tetrachlorodibenzo-*p*-dioxin. J Natl Cancer Inst.

[b24-ehp-118-1235] Thomaseth K, Salvan A (1998). Estimation of occupational exposure to 2,3,7,8-tetrachlorodibenzo-*p*-dioxin using a minimal physiologic toxicokinetic model. Environ Health Perspect.

[b25-ehp-118-1235] Turyk M, Anderson H, Knobeloch L, Imm P, Persky V (2009). Organochlorine exposure and incidence of diabetes in a cohort of Great Lakes sport fish consumers. Environ Health Perspect.

[b26-ehp-118-1235] Vasseur P, Cossu-Leguille C (2006). Linking molecular interactions to consequent effects of persistent organic pollutants (POPs) upon populations. Chemosphere.

[b27-ehp-118-1235] Welshons WV, Thayer KA, Judy BM, Taylor JA, Curran EM, vom Saal FS (2003). Large effects from small exposures. I. Mechanisms for endocrine-disrupting chemicals with estrogenic activity. Environ Health Perspect.

[b28-ehp-118-1235] Wolff MS, Camann D, Gammon M, Stellman SD (1997). Proposed PCB congener groupings for epidemiological studies. Environ Health Perspect.

